# Neurological involvement in the respiratory manifestations of COVID-19 patients

**DOI:** 10.18632/aging.202665

**Published:** 2021-02-14

**Authors:** Bai-Hong Tan, Jia-Mei Liu, Yue Gui, Shuang Wu, Jia-Le Suo, Yan-Chao Li

**Affiliations:** 1 Laboratory Teaching Center of Basic Medicine, Norman Bethune Health Science Center of Jilin University, Jilin, China; 2 Department of Histology and Embryology, College of Basic Medical Sciences, Norman Bethune College of Medicine, Jilin University, Changchun, Jilin, China

**Keywords:** respiratory failure, coronavirus, SARS-CoV-2, COVID-19, nervous system

## Abstract

The peculiar features of coronavirus disease 2019 (COVID-19), caused by Severe Acute Respiratory Syndrome Coronavirus-2 (SARS-CoV-2), are challenging the current biological knowledge. Early in Feb, 2020, we suggested that SARS-CoV-2 may possess neuroinvasive potential similar to that of many other coronaviruses. Since then, a variety of neurological manifestations have been associated with SARS-CoV-2 infection, which was supported in some patients with neuroimaging and/or cerebrospinal fluid tests. To date, at least 27 autopsy studies on the brains of COVID-19 patients can be retrieved through PubMed/MEDLINE, among which neuropathological alterations were observed in the brainstem in 78 of 134 examined patients, and SARS-CoV-2 nucleic acid and viral proteins were detected in the brainstem in 16/49 (32.7%) and 18/71 (25.3%) cases, respectively. To shed some light on the peculiar respiratory manifestations of COVID-19 patients, this review assessed the existing evidence about the neurogenic mechanism underlying the respiratory failure induced by SARS-CoV-2 infection. Acknowledging the neurological involvement has important guiding significance for the prevention, treatment, and prognosis of SARS-CoV-2 infection.

## INTRODUCTION

Since the beginning of 2020, a newly emerging coronavirus (CoV), known as Severe Acute Respiratory Syndrome CoV-2 (SARS-CoV-2), has spread rapidly among human beings all over the world, leading to a disease called coronavirus disease 2019 (COVID-19). SARS-CoV-2 not only causes acute, highly lethal pneumonia, but also infects many other systems, including the immune, cardiovascular, digestive, urinary, and nervous systems.

**Figure 1:**
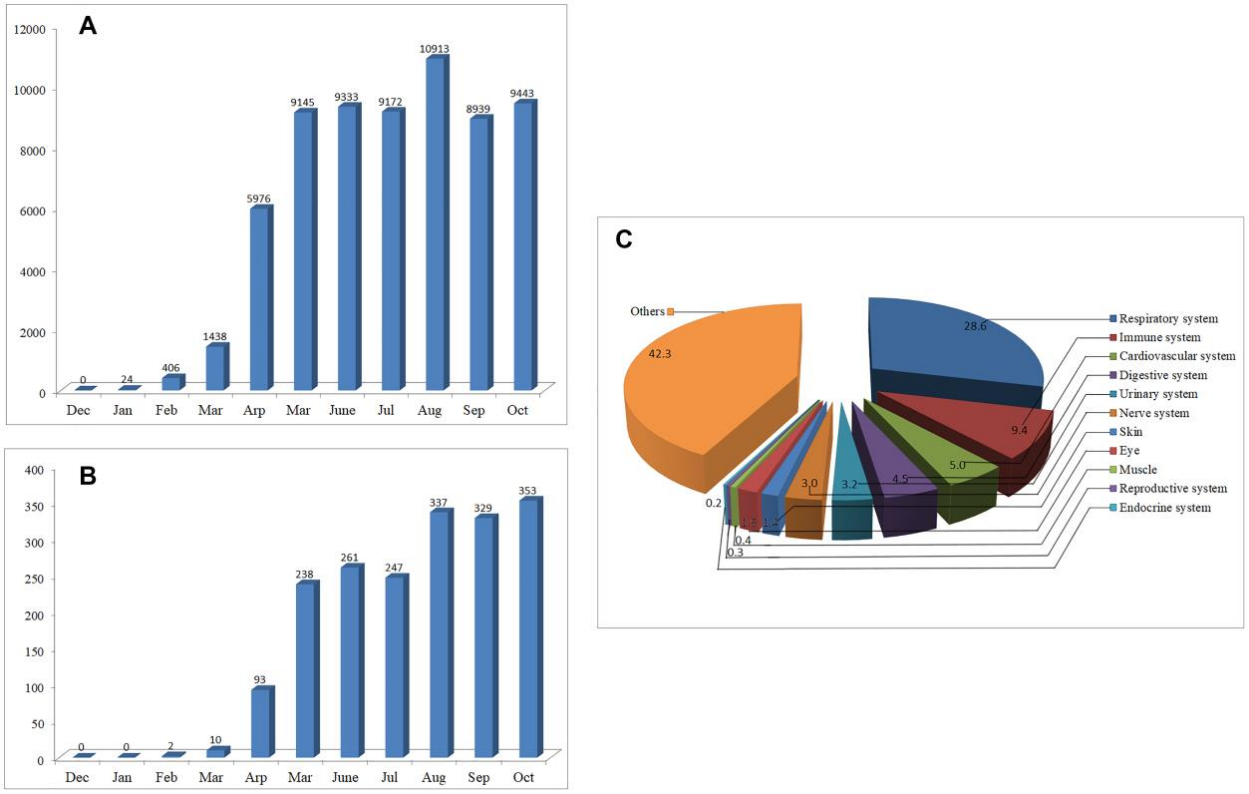
**The number of articles on COVID-19 published from Dec 01, 2019 to Oct 31, 2020.** (**A**) Monthly changes of the number of articles on COVID-19. (**B**) Monthly changes of the number of articles on COVID-19 neurology. (**C**) Percentage of the published articles on different systems of the body.

As of Jan 2020, only 24 articles on COVID-19 can be found through PubMed/MEDLINE, however, the number of papers increased exponentially over the next few months (Figure 1A). By Oct 31, 2020, over 64,000 articles on COVID-19 can be retrieved, indicating that the pandemic of COVID-19 has aroused great public concerns. Among the published data, 57.7% are related to organ involvement. The papers on the respiratory, immune, cardiovascular, digestive, urinary and nerve systems account for 28.6%, 9.4%, 5.0%, 4.5%, 3.2% and 3.0%, respectively. The remaining 42.3% focus mainly on disease prevention and treatment, virus structure, vaccines, epidemiological characteristics and so on (Figure 1B).

To provide a clue for the prevention, treatment, or further study of COVID-19, we suggested early in Feb 2020 that SARS-CoV-2 may have similar neuroinvasive potential to that of many other CoVs [1, 2]. In Feb 2020, only two articles can be found on the neurology or neuroscience of COVID-19. However, by Oct 31, 2020, more than 1900 articles on this topic can be retrieved, most of which were published after Apr 2020 (Figure 1C).

Neurological involvement in COVID-19, now called “COVID-19 neuroscience” or “COVID-19 neurology”, has attracted more and more attention [3, 4]. Elucidating the underlying mechanisms assist in formulating effective treatment strategies to reduce the mortality of SARS-CoV-2 infection. In this paper, we review the existing evidence regarding SARS-CoV-2 neuroinvasion and further explore its possible implications in the respiratory manifestations of COVID-19 patients.

## Evidence for the neuroinvasion of SARS-CoV-2

To date, a variety of neurological manifestations have been documented after SARS-CoV-2 infection. Neurological involvement in some patients were supported by neuroimaging findings [5–8] and positive detection of SARS-CoV-2 RNA in cerebrospinal fluid (CSF) [9–30]. Moreover, SARS-CoV-2 RNA and/or viral proteins were detected in the brains of some patients who died from COVID-19 [31–37].

The first-hand clinical report on neurological manifestations associated with SARS-CoV-2 infection was available as a preprint in medRxiv early in Feb, 2020, which was then published in *JAMA* in Apr, 2020 [5]. According to this report, 36.4% of the patients presented various neurological manifestations. Thereafter, Romero-Sánchez et al. evaluated 841 COVID-19 patients in Spain and found that 57.4% exhibited various neurological symptoms [6]. Pinna et al. analyzed the clinical records of 650 COVID-19 patients in Chicago, USA, and found that 7.7% of the patients showed neurological symptoms [7]. Similarly, Karadaş et al. evaluated 239 consecutive inpatients with COVID-19 in Ankara, Turkey, and detected neurological symptoms in 83 (34.7%) patients [8].

The COVID-19-associated neurological symptoms can be classified into three categories: 1) central nervous system (CNS) involvement, including headache, dizziness, consciousness disorder, epilepsy, and acute cerebrovascular accidents; 2) peripheral nervous system (PNS) involvement, such as olfactory loss, hypogeusia, visual impairment, and neuralgia, and 3) skeletal muscle injury [38]. Agarwal et al. analyzed the clinical data of 404 patients with COVID-19 in Washington, USA [39], and found that the most common CNS involvement was impaired consciousness (21.3%), followed by headache (20.3%) and dizziness (7.7%). The most common PNS involvement was muscle pain (32.4%), followed by the disorders of taste (6.7%) and smell (4.5%). Approximately 24.5% of COVID-19 patients showed acute neurological symptoms, of which the most common was mental state changes (21.3%), followed by critical illness myopathy (2.0%), stroke (0.7%), and seizures (0.5%) [39].

Of interest, neurological manifestations were reported to be the initial or the only symptom in many patients with COVID-19 [13, 24, 40–42], indicating that the nervous system may be one of the primary targets of SARS-CoV-2. Many neurological manifestations, especially those reported in critical patients with COVID-19, may be attributed to systemic inflammatory responses, hypoxemia, or multi-organ failure [43]. However, some special neurological manifestations, such as encephalitis, anosmia and hyposmia, may be related to the direct invasion of SARS-CoV-2 into the CNS.

Since Moriguchi et al. [24] and Xiang et al. [30] provided the first evidence of SARS-CoV-2 in the CSF of COVID-19 patients, so far, at least 26 cases have been reported to show positive CSF detection of SARS-CoV-2 [9–30]. Interestingly, in some cases SARS-CoV-2 RNA was detected in the neural tissues, but not in the CSF [31, 36]. Since the CSF test is related to the time of CSF collection, the severity of infection, or the sensitivity of detection methods, the negative outcomes of CSF tests are not equated with the absence of SARS-CoV-2 in the CNS [43–45].

Previous studies on some other neurotropic viruses show that invasion of viruses into the CNS is associated with the increase of intrathecal antibodies in the CSF [46–47]. Song et al. analyzed CSF samples from 6 COVID-19 patients, including 3 with encephalopathy, 2 with intractable headache, and 1 with seizures [48]. Strikingly, antibodies specific for SARS-CoV-2 were observed in the CSF in all patients. Using an animal model expressing human angiotensin-converting enzyme 2 (ACE2), they further found that the antibodies appeared or increased in the CSF only when the CNS was infected. As a matter of fact, antibodies specific for SARS-CoV-2 have been found in the CSF in 30 patients with COVID-19 in 6 case/case series reports [26, 49–53]. According to the results reported by Song et al. [48], the anti-SARS-CoV-2 antibodies in the CSF in patients with intact blood-brain barrier are closely related to the direct invasion of the virus into the CNS.

In support of SARS-CoV-2 neuroinvasion, Paniz-Mondolfi et al. reported the first autopsy evidence of the presence of SARS-CoV-2 in the brain of one COVID-19 patient on Apr 21, 2020 [31]. Since then, more and more autopsy studies show that SARS-CoV-2 can enter the CNS and infect a variety of brain regions [32–37]. To date, SARS-CoV-2 RNA and/or viral proteins have been detected in the olfactory mucosa/nerve/bulb [32–33], trigeminal ganglion [32–33], medulla oblongata [32, 37], cerebrum [35], and cerebellum [36].

Consistent with the hypoxemic and hypercoagulable state in most decreased patients, cerebrovascular accidents [35, 48, 54–59] and/or hypoxic lesions [54, 56, 58, 60–62] have been widely observed in the brain in COVID-19 patients. However, these are not contradictory to the neuroinvasion of SARS-CoV-2, since SARS-CoV-2 RNA and/or viral proteins were detected in the brains of patients with cerebrovascular diseases and/or hypoxic injury [37, 48, 54, 56, 59–60]. In addition, many autopsy studies observed severe microgliosis and/or lymphocytic infiltration in specific brain regions [35, 54, 58], especially in the brainstem [37, 54, 56–57, 62–63].

The extensive presence of SARS-CoV-2 in the CNS, as well as the distinctly different neuropathological changes, is well consistent with the broad spectrum of neurological dysfunctions documented in patients with COVID-19.

## The neuroinvasion of SARS-CoV-2 is associated with respiratory manifestations in COVID-19 patients

Respiratory failure is a major cause of high mortality induced by SARS-CoV-2 infection [64]. Approximately 10% of COVID-19 patients who developed respiratory failure had to be transferred to intensive care unit (ICU) for ventilatory support, and up to 79% of them died [65–66]. Therefore, clarifying the underlying mechanism is urgently needed to make a reasonable treatment plan to save patients' lives.

Based on the clinical and experimental data available for CoVs, we previously suggested that the neuroinvasive potential of SARS-CoV-2 may play a role in the acute respiratory failure of some COVID-19 patients [1, 2]. In this section, we discuss the peculiar respiratory manifestations of COVID-19 patients and further assess the existing evidence of the neurological involvement in the respiratory failure induced by SARS-CoV-2 infection.

### Lung injury alone cannot explain the respiratory performance of all the patients with COVID-19

Radiographic studies show that most hospitalized patients with COVID-19 showed bilateral multiple peripheral ground-glass opacities at chest computerized tomography (CT) examination [65, 67]. The development of lung lesions on chest CT is generally consistent with the clinical time course of COVID-19 progression [68].

According to a study by Wang et al., the extent of lung lesions was similar in all 138 COVID-19 patients, whether mild or severe [69]. This finding was further confirmed by several other studies [65, 70–71]. Surprisingly, asymptomatic patients were reported to show similar imaging abnormalities without a significant difference from those in symptomatic patients [72].

Despite obvious lung abnormalities, most COVID-19 patients showed only mild flu-like symptoms. Approximately 37.8 ~ 67.8% of patients presented cough [73–74, 71], but most of them did not have sputum production. The productive cough was present only in 12.1 ~ 35.9% of mild patients and 22.2 ~ 48.8% of severe patients [69, 73–75]. However, hypoxemia may develop in both mild and severe patients with COVID-19 [70, 76–77]. Of interest, 29.4 ~ 62.4% of severe patients and 74.4 ~ 84.9% of mild patients did not present dyspnea [73–75].

Many patients with SARS-CoV-2 infection came to hospitals with severe hypoxemia so that they should have lost consciousness or be close to organ failure. Surprisingly, they denied any difficulty with breathing, and showed no signs of using auxiliary respiratory muscles. This unusual clinical presentation has been termed as “silent hypoxia”, and is defying the current basic biology [78]. In some COVID-19 patients, the “silent” hypoxemia might last for a long time after receiving symptomatic support treatment, which gave medical staff an illusion of improvement. However, hypoxemia in some cases suddenly progressed and worsened from 10 ~ 14 days after infection so that these patients rapidly developed acute respiratory distress syndrome, respiratory failure, multiple organ failure and even death [65].

More than half of the patients with dyspnea are bound to develop to severe cases requiring intensive care [65, 67, 69]. However, many critical patients failed early attempts at weaning from invasive mechanical ventilation so that the time of ICU stay appeared to be very long [65, 79]. This is surprising since most of them have recovered from pneumonia.

Several researchers also noticed that more than 50% of ICU patients exhibited dissociation between the mechanical characteristics of the respiratory system and the severity of hypoxemia [80–81]. In these patients, the compliance of the respiratory system and the amount of gas in the lung were both in the normal range. This is strange and has rarely been reported in other forms of acute respiratory distress syndrome [80–81, 82].

Respiratory viral infection can cause inflammatory changes and stimulate the sensory receptors located in the respiratory system, and hypoxemia can stimulate the glomus cells in the carotid and aortic bodies. The resultant impulses in these sensory structures are transmitted to and processed through the respiratory center located in the brainstem. The accommodative demands from the brainstem are then transmitted down to the phrenic nerves and diaphragm and cause increased ventilation. Meanwhile, the enhanced activity of respiratory center is transmitted up to the cerebral cortex, producing a subjective feeling of shortness of breath [83–84]. As an important warning signal of self-awareness, the incidence of dyspnea is significantly lower in COVID-19 patients than that in patients infected with many other respiratory viruses, such as Middle East Respiratory Syndrome (MERS)-CoV (69%), respiratory syncytial virus (95%), and influenza virus (82%) [85–86].

Autopsy studies show that the lungs of COVID-19 patients are characterized by diffuse alveolar damage with hyaline membrane formation, pneumocyte activation, microvascular thrombi, lymphocytic inflammation, and proteinaceous edema [87–88]. The exudation and fibrosis in terminal bronchioles and alveolar walls may lead to poor diffusion of oxygen across the alveolar barrier, while the increased thrombogenesis in pulmonary microvessels may aggravate hypoxemia. However, no evidence shows that these changes can cause blunting of dyspnea [84]. According to a case series study reported by Guan et al., among 1099 COVID-19 patients requiring hospital care or ICU admission, 23% and 12% had normal chest radiographic observations, respectively [74]. Moreover, some patients with acute respiratory distress did not show any evidence of pulmonary thromboembolism [23]. These data indicate that the acute respiratory failure induced by SARS-CoV-2 infection cannot be explained only by the pulmonary changes [89].

Respiratory failure may be caused by disturbance of any part of the respiratory movement, including the respiratory center, nerves, muscles, thorax, airways, and lungs. As discussed in detail below, increasing evidence shows that either or both PNS and CNS are involved in the respiratory failure of COVID-19 patients.

### Neuromuscular dysfunction is associated with respiratory manifestations in some COVID-19 patients

Involvement of the PNS after SARS-CoV-2 infection includes anosmia, dysgeusia, Guillain-Barré Syndrome (GBS), myasthenia gravis myositis, myalgia, rhabdomyolysis, muscle wasting, and critical-ill myopathy [4, 43]. Rifino et al. performed a retrospective study on 137 COVID-19 patients with neurologic manifestations, and found that patients with PNS involvement more frequently developed severe acute respiratory distress syndrome compared to patients with altered mental status or cerebrovascular disease [26]. There exists strong evidence supporting that respiratory nerves and/or muscles are involved in the acute respiratory failure of COVID-19 patients.

The diaphragm is the main inspiratory muscle, whose abnormalities affect coughing and expectoration, and result in a significant decrease in respiratory volume [90]. Phrenic nerves originate from spinal motoneurons at the levels of C3 ~ C5, which are regulated by spinal descending pathways crossing the levels of C1 ~ C2. Damage to the neural circuit controlling the diaphragm causes rapid deterioration of respiratory mechanics [91]. In support of this, Maurier et al. reported a 58-year-old female with COVID-19 who showed fever, dysgeusia and anosmia at the onset and rapidly developed progressive dyspnea due to phrenic paralysis [92]. Of note, this patient did not show any cardiac, pleural, parenchymal or pulmonary abnormalities, and creatinine phosphate kinase levels were also normal. Borroni *et al*. reported two COVID-19 patients with focal diaphragmatic myoclonus [93]. Electroencephalogram (EEG) showed no structural damage in the CNS in case 1, but revealed lateralized periodic discharges in the brain in case 2. Interestingly, the periodic discharges were closely correlated with the diaphragmatic myoclonic movements in this patient.

Diaphragmatic weakness has been widely described in COVID-19 patients with GBS, among whom quite a few developed severe respiratory failure [94]. Rajdev et al. reported a 36-year-old man who was diagnosed with COVID-19-associated GBS [95]. Although chest imaging showed that the lung lesions were recovering, he developed acute respiratory failure due to neuromuscular weakness caused by bulbar palsy. Patients with GBS usually have concomitant diaphragmatic weakness, which leads to atelectasis in the base of the lung, resulting in decreased lung compliance and increased intrapulmonary shunt. These changes, together with pulmonary infection, might induce a severe decline in lung volume and rapid deterioration of hypoxemia in COVID-19 patients [94].

The diaphragm was also frequently affected in ICU patients due to critical illness and mechanical ventilation [96–97]. Although required for many patients with acute respiratory failure, invasive mechanical ventilation can partially or completely unload respiratory muscles and silence the respiratory centers in the brainstem, leading to the inactivity of the diaphragm [98–99].

To date, a large amount of clinical data show that SARS-CoV-2 infection is often associated with acute neuromuscular dysfunction [5, 38]. Furthermore, neuromuscular dysfunction has been reported to be an important cause of acute respiratory distress syndrome in COVID-19 patients with minimal chest imaging findings [100].

### Damage to the respiratory-related neural loops is associated with respiratory manifestations in some COVID-19 patients

Anosmia and dysgeusia are the most common PNS symptoms [43], indicating that SARS-CoV-2 infection may reduce the sensitivity of chemosensory reflexes [101–102]. Carotid/aortic bodies and bronchopulmonary C-fibers play a pivotal role in monitoring CO_2_, H^+^ and O_2_^+^ in the blood, and therefore damage to these structures has been suggested to be responsible for the absence of the sensation of dyspnea [103].

Carotid and aortic bodies are specialized sensory structures in arteries, where the cellular receptor for SARS-CoV-2, ACE2, is also present [104]. SARS-CoV-2 may directly invade the glomus cells in the carotid and aortic bodies or indirectly damage their sensory function due to the systemic inflammatory response and/or hypercoagulable condition in the blood. However, less than 1% of COVID-19 patients exhibited a detectable level of SARS-CoV-2 in the blood [105, 106], and the infection of carotid/aortic bodies has not yet been confirmed [58].

The affection of bronchopulmonary C-fibers has previously been reported to contribute to the respiratory failure induced by other respiratory viruses by abrogating the sensory transmission from lungs and respiratory airways [103, 107–108]. However, it is unclear whether this happens during SARS-CoV-2 infection.

Mechano- and chemoreceptors play a monitoring role in the lung and lower respiratory airways, while the respiratory reflex is triggered and controlled primarily by the respiratory center located in the brainstem. The brainstem is comprised of many important structures, which are essential for breathing, heart rate, blood pressure control, digestion, etc. These anatomical connections make the brainstem an easily accessible CNS target for SARS-CoV-2 from peripheral infection sites [1, 109]. In support of this, Lukiw et al. reported that the expression level of ACE2 was the highest in brainstem among 21 different brain regions in humans [110].

The brainstem has been reported to be highly infected with SARS-CoV [111] and MERS-COV [112]. In animal experiments, SARS-CoV and human CoV OC43 have been shown to enter the olfactory bulb after exposure to the nasal route and subsequently invade the CNS, including the brainstem [111, 113]. Considering the high similarity between SARS-CoV and SARS-CoV-2, we previously proposed that the potential infection of the brainstem may play a role in the acute respiratory failure of patients with COVID-19 [1–2].

### Clinical evidence for involvement of the brainstem in COVID-19

In line with possible involvement of the brainstem, a case series study on COVID-19 patients younger than 18 years with neurological symptoms reported brainstem signs such as dysarthria or dysphagia in 2 cases (2/18) [114]. Similar findings were reported in some old patients [32, 35, 37]. However, absent or impaired brainstem reflexes are more common in severe patients who have been diagnosed with COVID-19-associated encephalomyelitis or encephalopathy [28, 50, 115].

Involvement of the brainstem was supported with neuroimaging findings in some COVID-19 patients. Wong et al. reported a 40-year-old man in England, who developed acute brainstem dysfunction after SARS-CoV-2 infection [116]. MRI scans revealed inflammatory changes in the brainstem in this case. Virhammar et al. described a 55-year-old female with acute necrotizing encephalopathy [28]. The CSF sample from this patient was positive for SARS-CoV-2. MRI scans revealed abnormal changes in several brain regions, including the brainstem. To date, abnormal imaging changes of the brainstem have been widely documented in COVID-19 patients with GBS [117], necrotizing encephalopathy [10, 28], and encephalomyelitis [118–121].

As stated earlier, it is difficult for many ICU patients with COVID-19 to withdraw invasive mechanical ventilation, even if their pulmonary infections have recovered [65, 80–81]. Related to this, Koutroumanidis et al. found that 5 of 13 ICU patients with COVID-19-associated encephalopathy had alpha coma EEG pattern [122]. Alpha coma is typically associated with the lesions located in the brainstem reticular formation [123]. Therefore, the relatively high incidence of alpha coma in severe patients with COVID-19 indicates that brainstem injury may be an important reason why they were difficult to get rid of invasive mechanical ventilation [122].

As a possible mechanism, the nerve endings within the olfactory neuroepithelium have been considered an entry point for SARS-CoV-2 to infect the brainstem [106]. Consistently, the mechanism underlying COVID-19-related olfactory dysfunction was reported to be obviously different from patients in acute colds, and may reflect, at least to some extent, a specific involvement at the level of CNS [124].

Eliezer et al. reported a female with COVID-19 who presented an acute loss of olfactory function without nasal obstruction. In this patient, CT and MRI analysis showed bilateral inflammatory obstruction in the olfactory clefts [125]. In a postmortem brain MRI study, Coolen et al. reported asymmetric olfactory bulbs in 4 of 19 patients with COVID-19 [126].

In a retrospective cohort study, Lin et al. reported that among 51 COVID-19 patients with MRI examinations 26 (51%) displayed acute or subacute findings in the CNS, including cranial nerve abnormalities (6) and critical illness-associated microbleeds (3). Of note, four patients displayed abnormally increased olfactory bulb signals suggesting olfactory neuritis, which might be related to the anosmia experienced by these patients [127].

In a prospective study, Lu et al. used MRI to evaluate the brains of 60 patients who had recovered from SARS-CoV-2 infection, and found that the volume of olfactory cortices was significantly increased in these patients [52]. Of note, 41 patients (68.33%) showed neurological symptoms during SARS-CoV-2 infection, and 30 (50%) still had neurological symptoms even though they had recovered 3 months after infection.

### Autopsy evidence for involvement of the brainstem in COVID-19

Convincing evidence for involvement of the brainstem after SARS-CoV-2 infection has recently been reported in postmortem studies [44]. Among the published autopsy studies, neuropathological alterations were observed in the brainstem in 78 of 134 examined patients, including 18 with vascular accidents in the brainstem [54–55, 58, 128, 129–130], 15 with hypoxic injury in the brainstem [37, 54, 56, 63], and 65 with microgliosis/lymphocytic infiltration in the brainstem [36–37, 56–57, 62–63]. Among these cases, some had two or more types of these neuropathological changes. SARS-CoV-2 RNA and viral proteins were detected in the brainstem in 16/49 (32.7%) and 18/71 (25.3%) cases, respectively. The positive detection of SARS-CoV-2 was much higher in patients who showed microgliosis and/or lymphocytic infiltration in the brainstem, relative to patients with vascular accidents or hypoxemic lesions in the brainstem [32, 37, 54, 56, 60–61, 131].

The first autopsy study on the brainstem was published online on May 13, 2020 by Bulfamante et al. [131]. In this study, a 54-year-old man who died from COVID-19 was observed to have severe degeneration in the neural tissues along the pathway from the olfactory nerve to the brainstem. Thereafter, von Weyhern et al. performed a more detailed postmortem study on the brainstem in 6 COVID-19 patients in Apr, 2020 [63], and they found that neuronal degeneration was extensively present in the brainstem in all 4 examined cases. Although no detection of SARS-CoV-2 was performed in the neural tissues in the two studies, the predominant involvement of the brainstem could not be attributed to only hypoxemia or hemorrhages.

Direct invasion of SARS-CoV-2 into the brainstem was reported in three autopsy studies in Jun, 2020 [32, 60–61]. Meinhardt et al. performed an autopsy study on 32 COVID-19 patients in Berlin, Germany, and detected SARS-CoV-2 RNA in the brainstem in 4 of 23 cases [32]. Menter et al. performed an autopsy study on 21 COVID-19 patients in Switzerland, and found SARS-CoV-2 RNA in the brainstem in all 4 cases examined [60]. Solomon et al. performed an autopsy study on 18 patients in Boston, USA, and found SARS-RNA in the brainstem in 3 of 18 cases [61].

On Oct 5, Matschke et al. published an autopsy study on the brainstem in 43 COVID-19 patients in *Lancet Neurology* [37]. Among the patients, 37 (86%) showed abnormal changes, including astrogliosis and/or microgliosis, in all assessed brain regions, but microglial activation and lymphocytic infiltration were the most severe in the brainstem and cerebellum. SARS-CoV-2 RNA was detected in the brains of 21 (53%) of 40 tested patients including the brainstem from 4 patients. Immunohistochemical staining revealed that the nucleocapsid protein of SARS-CoV-2 was present in neuron-like cells in the medulla oblongata and in the cranial nerves which originated from the lower brainstem.

In several studies, the presence of SARS-CoV-2 in the CNS was confirmed with different detection techniques [31–32, 37]. However, in some cases, positive results obtained by PCR tests could not be corroborated with *in situ* hybridization or immunohistochemistry using the same samples [54, 61]. Interestingly, in quite a few patients with negative PCR tests, SARS-CoV-2 was detectable in the same brain areas with immunohistochemistry [37].

The brainstem infection with SARS-CoV-2 is also supported with animal experiments. Deer mice, the most studied and abundant mammals in North America, are susceptible to SARS-CoV-2 infection because their ACE2 receptor shares 17 of the 20 critical residues for SARS-CoV-2 binding. As reported in COVID-19 patients, intranasal inoculation with SARS-CoV-2 caused respiratory, digestive and neurological infections in deer mice [132]. In the CNS of infected deer mice, SARS-CoV-2 antigen has been detected in a variety of brain areas, including the olfactory bulb and brainstem.

Therefore, both autopsy and animal studies indicate that the brainstem is one of the primary CNS targets of SARS-CoV-2, which may be the dominant reason for the unusually rapidly deteriorative respiratory function in some COVID-19 patients. The evidence currently available shows that SARS-CoV-2 can invade the brainstem in a retrograde manner via multiple nerve routes, including the olfactory, trigeminal, glossopharyngeal, and vagus nerves [37, 132].

## The significance of acknowledging the neuroinvasive potential of SARS-CoV-2

Ever since their discovery in the late 1960s, the ability of CoVs to infect humans had been neglected by the international medical community [133]. Although two unexpected COVID pandemics, triggered by SARS-CoV and MERS-CoV, respectively, recall people’s interest in CoV-infections, the neuroinvasive propensity of CoVs has still not attracted enough attention over the last 20 years. Unlike SARS-CoV and MERS-CoV, the rapid global spread of SARS-CoV-2 has posed an urgent and serious threat to public health.

As the counterpart of SARS-CoV-2, the pathogenesis of SARS-CoV infection remains poorly understood [134]. Similarly, a comprehensive understanding of SARS-CoV-2 is also lacking. Therefore, understanding the neuroinvasive potential of SARS-CoV-2 is of great significance for the prevention, treatment and prognosis of SARS-CoV-2 infection.

During the outbreak of COVID-19, the mortality of ICU patients with neurological problems was reported to be higher than that of patients without neurological symptoms [135]. Similarly, experimental studies show that the neuroinvasion of SARS-CoV-2 dramatically increased the mortality of infected animals [136]. Given this, SARS-CoV-2 infection in human beings should not be allowed to develop without treatment. As an example of the opposite, it has been reported that two-thirds of ICU patients in some hospitals were directly admitted from home [137].

Vaccination is considered the best option. However, before effective vaccines are available, wearing masks is undoubtedly a simple and effective measure against SARS-CoV-2 transmission [138], since it protects against invasion of the virus into the CNS from the respiratory tract and lung.

It is known that the virus in neurons can escape from the surveillance of the immune system, especially at the early stage of infection. The initially infected neurons eventually become apoptotic or are cleared by immune cells, prior to which virus progeny may have spread to other healthy neurons. The trans-neuronal transmission of CoVs makes it difficult to completely eliminate the virus from the CNS [139]. Therefore, the CNS infection of SARS-CoV-2 should be given antiviral treatment as soon as possible.

Chloroquine and hydroxychloroquine, which have been clinically used in COVID-19 patients, were reported to exhibit limited CNS penetration [140]. These drugs interfere with the glycosylation of ACE2 and therefore disturb the interaction of ACE2 with the spike protein of SARS-CoV-2. In addition, they prevent the endocytosis and subsequent vesicular trafficking of SARS-CoV-2 by endosomal alkalization [141].

During the epidemic of COVID-19, some researchers noticed that the patients who had been treated with adamantanes did not develop clinical diseases [142]. In these patients, adamantanes were initially used to treat the underlying neurologic disorders such as multiple sclerosis and Parkinson's disease. Adamantanes are known to possess an antiviral capability by binding a pore formed by SARS-CoV protein E and by interfering with the lysosomal phase of SARS-CoV infection. Of note, they can penetrate the blood-brain barrier, therefore may be considered a candidate to protest against the replication of SARS-CoV-2 in the CNS.

Previous studies reported that inhibition of tubulin polymerization hindered the retrograde axonal transport of poliomyelitis virus along infected peripheral nerves [143]. Therefore, some microtubule-associated inhibitors that have the capacity of penetrating the blood-brain barrier may be considered candidates to inhibit SARS-CoV-2 infection in the nervous system [106].

After replication in the CNS, progeny virions were exocytosed from host neuronal cells, and entered the next-order neurons by endocytosis [144]. One of the treatment alternatives available for COVID-19 is administration of anti-SARS-CoV-2 antibodies in plasma. Of interest, previous studies on West Nile virus showed that neutralizing antibodies could prevent viruses from spreading from neuron to neuron [145–146].

To date, there are some candidate drugs that can be tested to stop the CNS infection of SARS-CoV-2 [140, 147]. According to action sites, the drugs against neurotropic viruses can be divided into at least four kinds: blocking the invasion, transportation, replication, and release of viruses, respectively. With respect to the neurotropism of SARS-CoV-2, the CNS penetration ability of drugs is a critical factor for the treatment of brain infection. However, it should be noted that a kind of antiviral drugs alone may not be enough to stop the infection. For example, inhibiting virus transportation cannot alter the redistribution and replication of viruses. Therefore, it is recommended to combine two or more antiviral drugs to interfere with different stages of the life circle of viruses in the CNS.

Experimental studies on HCoV-OC43 show that the presence of CoV RNAs might last for at least one year without being acutely toxic in the brains of infected mice that had survived [148]. This suggests that CoVs can establish a persistent infection within the CNS of their hosts, which significantly increases the risk of long-term disability [149]. Consistent with this hypothesis, neuropsychiatric or neurocognitive disorders have been reported in some patients who had recovered from acute SARS-CoV-2 infection [150]. It is noteworthy that the young Japanese man, who was confirmed as the first case of meningitis/encephalitis [24] and has recovered from COVID-19, was found to develop retrograde amnesia and cannot recall what happened to him during his own infection [151].

Since the impact of SARS-CoV-2 infection on the nervous system may last for a long time, it is necessary to follow up the neurological changes of discharged patients and develop appropriate neurorehabilitation measures.
